# Extra-Limites

**Published:** 1827-01

**Authors:** 


### EXTRA-LIMITES.

### I.

### MR. LIZARS' OBSERVATIONS ON LITHOTOMY.

TO

*The Editor of the Medico-Chirurgical Review of London.*

Sir,

Although, probably, more has been written on lithotomy than on any other surgical operation, yet, judging from the writings of our *most learned modern physicians and surgeons*, we seem to be still ignorant of its fundamental principles. I am led to these observations by two papers lately published, by Dr. Monro and Mr. Allan, in the 3d and 4th numbers of the New Edinburgh Journal of Medical Science, in which I am *obscurely* alluded to.

In the 3d number, Dr. Monro states, "Mr. Allan informed me, that he had met with an instance, in which there were two stones lodged within the bladder. One of these was readily removed by the forceps, but the other could not be extracted at the time; it, therefore, occurred that, as the groping for a considerable time with the forceps in the bladder proves extremely dangerous, it would, at the moment, be more prudent to desist from further attempts to extract the stone, which was then firmly grasped by the contracted portion of the bladder, and that the stone would probably come away of itself along with the urine, in consequence of the subsequent relaxation of the contracted part of the bladder."

In Number 4, the Doctor says, "since the publication of my observations on spasm of the passages for the food, the bile, and the urine, I have received the following *important* communication from Mr. Allan, surgeon, which affords a striking illustration of the spasmodic power of the bladder of urine. I was present at the operation to which Mr. Allan has alluded, in the performance of which, the dexterity of the operator was no less conspicuous than his coolness and sound judgment.

Important Communication.

'*Letter from* Robert Allan, Esq. F.R.S. E. *Surgeon to the Royal Infirmary, and Lecturer on Surgery in Edinburgh, to* Alexander Monro, M.D. F.R.S. E. &c.

'57, *York Place, June* 20*th*, 1826.

'DEAR SIR,

"You will no doubt *recollect* of having been *present*, ten years ago, at an operation of lithotomy, performed by a *young* surgeon, on which occasion I acted as HIS PRINCIPAL ASSISTANT. The bladder was soon cut into, and a stone of some magnitude readily extracted; but we were conscious of the presence of another stone, although the operator, after the repeated introduction of the forceps, failed to extract it. The difficulty of extraction arose from the bladder spasmodically contracting upon the stone, and thus preventing the forceps from coming into contact with it. I introduced my finger into the wound, and felt a stone above the pubis, *(pubes)* which was firmly held in this situation by the spasmodic contraction of the bladder. I could just reach the stone, and turn it round and round, but my finger was too short to dislodge it. As the *operator was not provided with a lever, or with curved forceps*, I ADVISED that the patient should not be kept longer upon the table, but that he should be put to bed, and that, as the opening was free when the spasm of the bladder was relaxed, and suppuration established, there was little doubt but that the stone would be easily extracted; whereas, by persisting in fruitless attempts to extract, the patient would be so much exhausted, that his danger would be thereby greatly increased. The patient was, therefore, laid in bed, and when I went to visit him, along with his surgeon, on the evening of the third day, I introduced my finger into the wound, and hooked out, with the utmost ease, a stone about the size of a turkey-bean, *(French or kidney bean, phaseolus)* which was lying in the inner opening. The patient recovered without one bad symptom, and *this operation I have frequently revolved in my mind, and kept the case in view, as a guide in similar circumstances.* Quære. When the incisions are free, and the *delivery* (extraction) of the stone prevented by the spasmodic contraction of the bladder, as this will not relax till the patient is exhausted, and brought into danger, *is it not the preferable practice to put the patient to bed, instead of employing force, or persisting in fruitless attempts at extraction?* I am. &c.

ROBERT ALLAN.'"

Now, Mr. Editor, what are we to think of the state of the surgical profession in this learned medical city, when we find the Professor of Anatomy and Surgery, and the *first operator* to the Royal Infirmary and *Lecturer* on *Surgery*, so ignorant of the history of lithotomy, as, from the preceding communications, we must hold them to be? What must these gentlemen have taught for upwards of twelve years to their pupils, and into what dilemmas must many of their pupils have been brought, when performing this operation? The practical rule of not attempting beyond a few minutes to extract a calculus, is laid down in most distinct terms by Celsus,* by Albucasis,† by Franco,‡ by Fabricius Hildanus.§ It appears unnecessary to give any more lengthened quotations than those we have inserted below; let it suffice merely to mention the other authorities,—Covillard, Obs. Iatro-chirurgique, Observ. IV; Colot, Traité de l'Operation de la Taille, p. 178, 182; Saviard, Nouveau Recueil d'Observations Chirurgicales, Observ. CVIII. also, Observ. XLIII.; Tolet, Traité de la Lithotomie, Chap. XVII. Louis, Journal de Medecine, T. LXXXI.; Haller, Bibliotheca Chir. Tom. II.; Mangetus, Bibliotheca Chir. Tom. I.; Heister's System of Surgery, Part 2, Sect. 5, Chap. 141; Camper, London Medical Journal, Vol. 10, Part 2, p. 182, 1789; Percy, Journal de Medecine, Tome LXXIX.; Deschamps, Traité de l'Operation de la Taille, Tome IV. Now, Mr. Editor, not one of these authors, with the exception of Celsus, is mentioned by Mr. Allan in his HISTORY of the operation of lithotomy in his folio work, entitled, "Treatise on the Operation of Lithotomy," 1808. Whether Mr. Allan means to arrogate to himself the

* At si plures calculi sunt, singuli protrahi debent: sic tamen, ut, si quis exiguus supererit, potius relinquatur, si quidem in vesicâ difficulter invenitur, inventusque celeriter effugit. Ita longâ inquisitione vesica læditur, excitatque inflammationes mortiferas; adeo ut quidam non secti, cum diu frustraque per digitos vesica esset agitata, decesserint. Quibus accedit etiam, quod exiguus calculus ad plagam urinâ postea promovente, excidit.—*Celsus, Liber* VII. *Caput* XXVI.

† Nec calculum extrahas, sæpe enim id perdit ægrotum; dein curato vulnus, et cum sanguinis post dies aliquot sedatur fervor, et locus pútrescit, ad opus tuum redi, donec calculum extraxeris.—*Albucasis, Liber* II. *Caput* LXI.

‡ M'etant quelquefois advenu, que après avoir tiré une pierre, le patient etoit tant debile, que je n'osoye plus entreprendre de le plus presser pour savoir s'il y en demeuroit point d'autres, craignant qu'il ne mourust entre mes mains. Or, ayant mis les appareils sur la playe, et bendé comme avons dit dessus je le l'essoye jusques à ce qu'il fust plus fort, et bien souvent ay trouvé que, en changeant le premier appareil, on apprest, que la pierre qui estoit demeurée estoit sortie du tout dehors d'elle même.—*Franco, Traité des Hernies, Chap.* XXXIII.

§ Atque ita consultius est, inquit, calculum frustulatim extrahere, quàm ægrum tanto dolore atque miseriâ opprimi, semperque è duobus malis minus est eligendum. Nullum addit dictus *Dn. Francus,* huc usque auctorem inveni, qui hoc modo lithotomium administrârit, sicuti reverâ multis mirum videtur, quòd æger, post factam incisionem, per quinque vel sex dies plus minùsve non attingendus, tum demumque calculi extractio instituenda sit.—*Fabricius Hildanus de Lith. Liber, Cap.* 16.

merit of this mode of operating by his *Quære*, or whether he is actually ignorant of the history of this operation, or both, requires no Œdipus to inform us. When I read these papers, I could scarcely believe that I was reading the conjoint production of two distinguished Professors.

That the operation, "en deux tems," should be always kept in view by the operator, I have invariably stated to my pupils, for in no other way can we, in many instances, save the lives of our patients. I conceive that we ought never to perform the "operation premeditée en deux tems," but that we should perform the "operation necessitée en deux tems," or combine both. We should, therefore, be prepared with a perfect knowledge of both, and commence with the premeditated intention of leaving the calculus in case of necessity.

Mr. Allan uses the innuendo phrase "a *young* surgeon," as if youth was any disparagement to an operator. I should recommend him to read Lord Chatham's reply to Mr. Walpole in 1740, every word of which will make his grey "hairs stand on end, like quills upon the fretful porcupine," unless he is *not* "made of the same stuff, and cast in the same mould as other men are." It would be well for Mr. Allan were he young, that he might have time to learn the history of his profession. "Youth is the worthier age, for young men see visions, while *old* men *only dream* dreams." The youths who have just finished their career in the dissecting-room ought to operate in half the time of those, who have neglected the use of the knife on the dead body for thirty years, and who obstinately continue to do so. "The surgeon just come from the dissecting-room, is competent to all operations. They are dissections of sound and healthy parts."

Mr. Allan, in the next place, has the generosity to state, the "operator was not provided with a lever, or with curved forceps." Now, Mr. Editor, was it not the duty of the "principal assistant," an office which Mr. Allan seems to dwell upon with feelings of pride, (for he had not then even performed lithotomy himself) to take care that every instrument was at hand before the operation was begun? With regard to his "*advice*, that the patient should not be kept longer upon the table, but that he should be put to bed," Mr. Allan surely does not mean the world to believe that *he* was the *first* who has given such an advice—he would not wish us to consider *him ignorant* that the same recommendation had been given by Celsus, and every author on lithotomy, with the exception of himself.*

* See Allan's Treatise on Lithotomy.

To crown this *correctly drawn-up case*, be it known to the medical and surgical world, that Dr. Monro, who has paid me the handsome compliment, that, "in the performance of the operation, the dexterity of the operator was no less conspicuous than his coolness and sound judgment"—did not witness the operation—I repeat, Mr. Editor, that Dr. Monro never saw me perform this or any other operation. He was no doubt invited to be present, but, after the hour appointed for the operation had expired, I commenced operating, (resolved never to sport with the feelings of a patient for the convenience of any gentleman) and had finished the operation, put my patient to bed, and had left the patient's house, when Dr. Monro and a young gentleman appeared on the road advancing towards me. So much for the reliance to be placed on Dr. Monro and Mr. Allan's surgical cases.

I shall now briefly mention that, within these few weeks past, I have been again compelled to perform lithotomy "en deux tems," and have deeply to regret that Mr. Allan was not present, either in the capacity of my "principal assistant," or one of my "advisers." His elegant dexterous hand, and his profound historical knowledge of this operation, would no doubt have been of prodigious service!

While on a professional visit to Brechin this summer, I was requested (on passing through Montrose) by Dr. Crab, to see a man who had been labouring under calculus for about six years, and who had been previously sounded by the Dr. so carefully, that he was confident of the presence of a calculus, and, therefore, requested me to operate (the Dr. himself having for some years past resigned the use of the knife). At the recommendation of the other medical gentlemen of Montrose, the patient was removed to the hospital for the benefit of better light during the operation, and of superior accommodation for the man. He was a tall, large-boned person, somewhat emaciated, and upwards of 60 years of age. His bowels were opened by laxative enemata, or rather the rectum was emptied by laxative enemata, and soon afterwards he was carried into the operating theatre, when, after he was secured in the ordinary way, I filled the urinary bladder with tepid water by means of a catheter, which instrument, both before the introduction of the water and afterwards, at once pointed out the presence of a stone. The catheter intended for this purpose should have an ox or pig's bladder mounted on the end, which may be termed the handle, in the same manner as is done with the common glyster-bag and pipe. The catheter should be inserted in the urinary bladder of the patient, with the pig's bladder in a flaccid state, and then the tepid water ought to be poured into the pig's bladder, which is to be tied and gently squeezed, so as to distend moderately the urinary bladder of the patient. After sufficient water has been injected, which can be judged of only by the feelings of the patient, the catheter is to be withdrawn, and a tape tied round the penis a little below the glans. This method, when the irritability of the bladder of the patient does not prevent it, is superior to that of tying the penis some hours before operating, in order to accumulate the urine.

The staff was now inserted in the urethra onwards to the bladder, and given to Dr. Campbell, one of the surgeons of the hospital, who kindly assisted me. I then made a long incision through the skin and cellular substance, from the root of the scrotum, between the raphe of the perineum, and the ramus of the os ischii downwards, or coccygead beyond the anus, and over the fibres of the gluteus maximus muscle. The second incision was more limited superiorly or pubic, in order to avoid wounding the accelerator urinæ and erector penis muscles, but equally extensive downwards or coccygead, so as to divide some of the fibres of the gluteus maximus muscle: in this second incision, the transversus perinæi muscle was divided. One or two scratches with the scalpel, held in the same manner, as when making the first external incision, (for there appears no necessity for turning the edge of the knife upwards or pubic to the symphysis pubis, as directed by lithotomists) easily enabled me to arrive at the membranous part of the urethra, and to divide the levator ani muscle from this downwards to the bottom or coccygeal aspect of the wound, carefully avoiding the rectum. The point of the scalpel was then entered into the groove of the staff (the latter of which was now held close up to the symphysis pubis, and at right angles to the axis of the brim of the pelvis,) and carried onwards so as to cut the membranous portion of the urethra, the prostate gland, and a portion of the urinary bladder, lateralizing the knife so as to pass between the termination of the ureter and vesiculæ seminales, and depressing the rectum with the middle finger of the left hand to prevent its being wounded.* The tepid water and urine flowing copiously from the wound, I inserted my left fore-finger into the bladder, laid aside the scalpel, and conducted the forceps into the bladder, and then withdrew the staff.

---

* The scalpel is much the preferable instrument for the performance of this operation, as all are or should be, familiar with its use on the dead body, before undertaking an operation such as lithotomy. If the operator considers it too short, let him have one with a longer handle. The man

I at once felt the calculus distinctly at the fundus of the bladder with my left fore-finger, and easily touched it with the forceps in their shut state, but whenever I opened them over the calculus so as to seize hold of it, I found that the blades grasped the soft bladder which retained the calculus in situ. I then tried a scoop, but with equal want of success. I regret for Mr. Allan's satisfaction, that I did not use a "*lever* or *curved forceps*." After several fruitless attempts for five minutes, I resigned the forceps to Dr. Campbell, and after he had persevered for three minutes longer, I requested him to desist. I next untied the patient, put him to bed, and desired him to have an opiate. I now begged the medical gentlemen present to return to the operating theatre, when I explained the nature of the "operation en deux tems," and stated, that when all action had subsided, and suppuration was fairly established, the urinary bladder would offer then no resistance, and that probably the calculus would be in the mouth of the wound, or at all events, that it would be easily reached and extracted.

The operation was performed at 12 o'clock on Thursday the 5th of October, 1826, and as I had to proceed onwards immediately to Edinburgh, I begged that the patient should be bled whenever reaction had taken place, being of opinion, that after lithotomy, as after the operation of trepanning, venesection should be performed. The same precautionary means ought to be employed in all great operations. I mentioned that the calculus ought to be removed on Sunday or Monday, or whenever suppuration was fairly established. On Monday the 9th, a calculus of an oval flattened shape, and weighing three ounces and a quarter, was extracted with the forceps, a sound being previously inserted in the urinary bladder. By the last account which I received the other day, the patient was walking about, and the urine flowing along the urethra, and the wound nearly healed.

I have been thus particular in describing this case, as the steps deviate a little from those recommended by Mr. Allan and other authors on Lithotomy. I am decidedly of opinion,

---

who uses Dubois' knife, or any other instrument has given up dissection, and is therefore unqualified to operate. I should recommend such surgeons, to confine themselves to the routine practice of prescribing a dose of salts. "The passion of inventing instruments so conspicuous of late years, originates with those who know not how to use the common instruments." In every dead body, with the exception of one, that either any of my pupils or myself have operated on for lithotomy, part of the vesiculæ seminales has been wounded. In one or two rare instances, the termination of the ureter has been cut. I believe that I may state, the operation has been done upwards of 100 times.

that had I persevered longer in attempting to extract the calculus at the time of the operation, I would have induced such a degree of inflammatory action, as would, in all probability, have destroyed my patient. For, as my late worthy preceptor, Mr. John Bell, observes, "I fear there are few modern operations free entirely from the cruelties imputed to the apparatus major"—which fact, I deeply regret to state, every student at this school too often witnesses.

Either after the first stage of the "operation en deux tems," or "en un tems," if we may use the phrase, I conceive that no flexible catheters or tubes or tents ought to be inserted in the wound, as practised by Colot, Le Dran, and others; for, as Mr. John Bell observes, "In the old way of lithotomy, they introduced a canula into the wound, ostensibly to prevent healing, but really because they could not heal the wound." If the external incisions are free, and no mangling has been inflicted, we have nothing to fear from the urine—no infiltration will take place. To show the absurdity of fearing such an event, let us consider what Le Raoues, a celebrated lithotomist, did, who operated by cutting on the gripe. He endeavoured *not* to make the external incision correspond with the internal one, in order that the integuments might act as a valve. And he plastered up the wound with eggs and flour. And he often cured his patients in five days.

I have already stated, that venesection should be practised after lithotomy, whenever reaction has taken place, for the purpose of preventing inflammation. But if this diseased action should take place, the blood-letting ought to be repeated to syncope, and if the patient is an adult, 50 or 60 leeches should be applied to the hypogastric region: if a boy of three or four years of age from 18 to 24 leeches should be applied, and the external jugular vein opened, if one in the arm cannot be selected, in consequence of its smallness. After the leeches have dropped off, the warm bath should be used, and if pain on pressing the hypogastric region still continues, a tobacco enema should be administered, and be repeated as often as necessity requires, and as soon as its nauseating and exhausting effects have disappeared. It ought to be regulated, so as not to produce vomiting. Besides the above remedies, diluent injections of warm milk and water should be used to the wound, together with anodyne poultices and fomentations, both to the wound and to the hypogastric region. The patient ought to be kept in the warm bath until a sensible depleting or exhausting effect is produced. Blisters may be also used after the leeches.

JOHN LIZARS.

*Edinburgh,* 33, *York Place,* 10*th Nov.* 1826.

### II.

### MEDICAL JURISPRUDENCE.

### MEDICAL DEGREES—ABERDEEN—St. ANDREWS.

We have much pleasure in giving publicity to the following regulations of Aberdeen and St. Andrews, against which so much declamatory abuse has lately been discharged. If these universities adhere punctually to the regulations now enacted, all reasonable cause for reproach will be removed.

" '*Marischal College and University, Aberdeen.—Regulations respecting Medical Degrees.*

" ' 1. Every Person offering himself as a Candidate for the degree of M.D. shall, 1st. Produce satisfactory evidence of his possessing a good character, and of his having attained the age of twenty-five years ; and, 2dly, shall lay before the Senatus Academicus, certificates of his having obtained the degree of A.M. in this or some other University, after the usual examinations ;* of his having attended courses of lectures on Anatomy, Surgery, Chemistry, Materia Medica, Theory and Practice of Physic, and Botany, in this or some other University, or celebrated School, under Professors or Teachers of reputation ; and of his having attended, for three or more years, a Medical Hospital containing the average number of at least eighty patients.

" ' 2. After the Senatus Academicus shall have been fully satisfied on the above preliminary points, the applicant shall be received as a candidate for the degree of M.D. ; and shall be required to appear before the University, at one of their stated terms for granting such degrees, and in their presence be examined, by the Medical and other Professors, on the different branches of Medical Science—on his knowledge of the Greek and Latin Languages—and on such other branches of literature as they shall see proper. If fully satisfied with the qualifications of the candidate, the University shall confer on him the degree which he solicits.

" ' By order of the Senatus Academicus

" ' John Cruikshank, *Secretary.*' "

" These are the only conditions upon which the degree of M.D. can now be obtained at Aberdeen ; and it is presumed that henceforth the graduates of the universities there may claim the same rank as those of Edinburgh and Glasgow. The author of the 'letter,' &c. will at least acknowledge them equal to those of Glasgow ; the regulations of their respective universities being *nearly* the same ; what difference there is between them, is decidedly in favour of Aberdeen, as on looking over the Glasgow regulations I see no provision for testing the candidate's general acquirements.

" *Staffordshire*, 21*st May*, 1826. Vindex."

" '*Regulations respecting Medical Degrees, appointed by the Senatus Academicus of the University of St. Andrews to be observed in all time coming.*

" ' 1st.—No degree shall be conferred on an absent candidate.

---

" * As this requisition, respecting the degree of A.M. might prove injurious to some Medical Students whose education is now in progress, were it to take effect immediately, it is resolved that it shall not come into full force till the year 1830."

" '2d.—The candidate must produce a satisfactory certificate that he is upwards of twenty-one years of age, and that he is of unexceptionable moral character.

" '3d.—The candidate, if he be not in possession of a diploma as A.M. must produce certificates of his having had a Liberal and Classical Education, and be ready to undergo an examination as to his proficiency in Classical Literature by a committee of the University, or by the Senatus Academicus.

" '4th—The candidate must produce certificates that he has attended in some University, or celebrated school of Medicine, for at least four complete sessions, during four years, the following branches of Medical Education, viz. Anatomy and Surgery; Practical Anatomy, or private Dissections; Materia Medica and Pharmacy; Chemistry and Chemical Pharmacy; the Theory of Physic; the Practice of Physic; Midwifery and the diseases of Women and Children; Botany, and Clinical Lectures delivered by Professors, or accredited Medical Officers, on the cases of the sick in a large public Hospital. (It is not meant that the candidate shall have attended more than two or three of the above-mentioned Medical Classes during one session.)

" '5th.—The candidate must bring certificates of his having attended the daily visitations of the Physicians and Surgeons of such Hospital for at least six months a-year during two different years.

" '6th.—If the candidate shall have so far satisfied the University, the rector shall be requested to fix a day for his examination by the Medical and other Professors on all the branches of medical science; and provided this examination shall satisfy the Senatus Academicus, the candidate shall have delivered to him a Medical case or cases with questions subjoined, which questions he must answer in writing, and defend his answers before the Members of the University.

" '7th.—The whole proceedings shall then be submitted to the consideration of the Senatus Academicus, and if they are satisfied, the degree of M.D. shall be conferred on the candidate by the rector in the hall of the public library.

" 'Such are the regulations appointed by the university of St. Andrews to be observed in all ordinary cases. When, however, the candidate, possessed of a Surgical Diploma from Edinburgh, London, Dublin, or Glasgow, shall have been in regular practice for some years, or when he shall have been appointed and have acted as surgeon in his Majesty's navy or army, or to the forces or ships of the East India Company, a three years' attendance on the above-mentioned courses of medicine, and one year's attendance on a public hospital, shall be deemed sufficient to rank him as entitled to be examined.

" 'Farther, it is desirable that the candidate communicate by letter with the Medical Professor, and send him a list of the certificates he means to produce before he repairs to St. Andrews for examination. This may prevent disappointment, delay, and expense to the candidate.

" '8th.—The university reserves to itself the power to confer honorary degrees in Medicine on highly distinguished individuals, who cannot be expected to comply with the above regulations; but every such honorary degree shall be conferred unsolicited and free of expense.

" '*St. Andrews, 25th February,* 1826.' "

### III.

### HUNTERIAN MUSEUM.

Before making any remarks of our own, we shall lay before the public the two following circulars, transmitted to us with permission to make what use of them we please.

### 1. *Dr. Ager's Circular.*

The London Medical and Physical Journal for September last contained a Letter from an anonymous Licentiate, complaining that the right of claiming admission, and of introducing Visitors to the Hunterian Museum, was restricted to the Fellows of the College of Physicians and the Members of the College of Surgeons; and proposing, that a remonstrance should be sent by the Licentiates to the *Trustees*, on the supposition, that they had made the regulation. The Editor of the Journal, Dr. Macleod, observed, "if this be correct, there can be but one opinion of the transaction;" and then demanded an explanation from the *Censors*. Having accidentally seen these remarks, I considered it my duty to point out the error at once to the President in a Letter, dated on the 13th of the same month. It was thought best to enable the Editor to correct the mistake through the medium of Dr. Chambers: who, on the 26th, wrote, that "Macleod wished to be entirely impartial on the subject of the Censors, and would have been very glad of any good answer to the Licentiate's letter;" and requested the Registrar, "if there appears to Sir Henry no objection to the publication of Dr. 'Ager's Remarks,' to send them in the course of the day for that purpose. This was communicated to me, at the President's desire, on the following morning, with a request, that I would send an explanation to the Editor as soon as possible, lest the reputation of the College should suffer from continual misrepresentations remaining uncontradicted. I therefore wrote immediately to Dr. Macleod, observing, that such explanation from the Censors appeared to me "hardly necessary; for surely every liberal and intelligent person would conclude, that the regulation did not proceed from them: however, I can have no objection to its being stated on my part, that it was one of the original conditions, comprised in a Treasury-Minute, when the Museum was presented to the College of Surgeons, which the Trustees were merely appointed to see enforced." This Letter was published in the Journal for October, preceded on the same page by farther strictures on the conduct of the Censors, asserting a belief, that their "officious interference with the College of Surgeons," is "condemned by none more heartily than by the members of their own body;" calling in question "their taste and judgment in interfering in the business at all;" and yet insisting, that if they could have procured for the Licentiates the right of admission, as they have not done so, "nothing that has been said of them is too severe, and nothing that we have said is severe enough." Having drawn up the following statement of facts, in the accuracy of which the two junior Censors concurred, I thought it proper to show it to Dr. Frampton, as the charge unjustly implicated him also. He at once declared, that he entirely approved of what had been done; and candidly added, that if he had not, it was his duty to have attended the meetings, and opposed it. He also agreed with us in the propriety of submitting the whole matter to the President, since an imputation was thrown upon the College rather than ourselves; and leaving it to his discretion to take any steps which he might think the interests of that body required on the occasion. This was accordingly done; and the President has replied through the medium of the Registrar, that "he thinks the statement perfectly satisfactory," and recommended its being made public. I have felt some difficulty about the best method to be adopted for that purpose. Of course, the London Medical and Physical Journal was entirely out of the question: but thinking it particularly important, that these transactions should be fully known to the Members of the College, I have determined to distribute a Circular Letter among them, and a few other friends.

JOSEPH AGER.

*Statement of Facts.*

When the Hunterian Museum was presented to the late Corporation of Surgeons, a Treasury-Minute of the Conditions was drawn up, and certain Trustees were appointed to see them enforced; among whom are the President and four Censors of the College of Physicians. The first condition is, that "the Collection shall be open four hours in the forenoon, two days every week, for inspection and consultation of the Fellows of the College of Physicians, the Members of the Company of Surgeons, and persons properly introduced by them; a Catalogue of the preparations, and a proper person to explain it, being at those times always in the room." The number of days of admission had been diminished, to give more time for proceeding with the Catalogue; and a general complaint was made among the Members of the College of Surgeons, that the Museum was not sufficiently open and useful to them.

At the Quarterly Meeting of the Trustees on the 6th of May last, there were present, the Duke of Somerset, Mr. Davies Gilbert, Dr. Ager, Dr. Elliotson, and Dr. Ramadge. The last Gentleman suggested to the Board the propriety of making the days of admission more frequent, in which all the other Trustees concurred; but it was thought advisable, that an extraordinary meeting should be called, and the Curators summoned to attend on the 20th May, to enable the Board to decide finally upon that subject, as well as the means of completing the Catalogue as soon as possible.

At this second meeting, besides the Trustees present at the preceding, Lord Colchester and Sir Everard Home attended; but the latter Gentleman withdrew on the introduction of the Curators. Arrangements were made for expediting the preparation of the Catalogue, and it was determined unanimously, that the original conditions should be strictly enforced.

At the next Quarterly Meeting, on the 5th of August, there assembled Lord St. Helens, Sir E. Home, Dr. Ager, and Dr. Ramadge. A communication was received from a Meeting of Members of the College of Surgeons, conveying their thanks for what had been already done; and requesting, that the times of admission to the Museum should be increased, and the Licentiates of the College of Physicians, as well as other respectable Medical and Scientific Persons, privileged to attend. These regulations had indeed been suggested to the Board by the Censors present at the former Meetings; but it was seen with regret, on referring to the original conditions, that the Trustees had no power to enforce them. The only arrangement then made respected the interpretation of the terms "properly introduced." A personal introduction had previously been required; but it was unanimously agreed, that a Letter should be thenceforth considered sufficient, and that the regulation thus interpreted should be communicated to those Gentlemen who had applied to the Board.

### 2. *Dr. M'Leod's Circular.*

*To* Sir HENRY HALFORD, Bart.

*Henrietta-street, Cavendish-square;*

SIR, *November* 10*th*, 1826.

Some public discussion having taken place with regard to the line of conduct adopted by the late Censors of the College of Physicians, in the questions which have recently been agitated in the College of Surgeons; and the part which I have taken on this occasion having been represented as disrespectful towards that body, of which I am a Licentiate, I take the liberty of soliciting your attention to the following observations.

On a recent occasion, a circumstance occurred at a meeting of the Trustees of the Hunterian Museum, by which the feelings of the Licentiates were hurt, as (till it was explained) it appeared to place in a conspicuous point of view the preference given to the Fellows. That the impression produced by this occurrence was of the nature I have stated, is well known; and several letters were addressed to me upon the subject, as Editor of a Medical Journal. One of these being more temperate than the others, I published, appending to it a note, in which I remarked that the letter proceeded upon the "assumption" that the Trustees had unlimited power to admit whom they pleased; adding, that I should like to hear what the Censors had to say in explanation. It is quite obvious that an opportunity was thus afforded to the Censors of conciliating the Licentiates, by explaining the circumstance which had given rise to the misunderstanding. A month, however, elapsed, but no communication was made; and it was not till the ensuing Number of the "Medical and Physical Journal" had been completed, and was on the eve of being struck off, that a letter was received,—not, indeed, from the Censors, but from one individual among them, who expressly stated it to be on his own part. This letter reflected on me as requiring what no "liberal and intelligent" person would have deemed necessary; but, as it seemed intended to explain the circumstance which had prevented the Licentiates from being, in this instance, placed on the same footing with the Fellows, I was induced to stop the press for its insertion; having stated in the preceding paragraph, that, if the Censors had not the power to grant what was desired, "no impartial person" would "blame them."

But, in the remarks alluded to, and which have given so much offence to the late Censors, I commented on the questionable policy of their interfering with the Hunterian Museum at all at such a time; because I regarded their co-operation with those surgeons whose avowed object was the abrogation of the Charter of their College, as calculated to embroil the two corporate bodies, and therefore as injudicious; as contrary to all established etiquette in the profession, and therefore as in bad taste. And, I ask, in the event of public meetings being held by the Licentiates to overthrow the authority, and destroy the Charter, of that learned body over which you preside,—I ask whether, under such circumstances, it would not be regarded as a most ungracious act, were the Council, or any other officers of the College of Surgeons, to avail themselves of any influence their official situations might accidentally give them, and to volunteer their services on the side of the Licentiates?

But, so far was I from wishing to implicate the College generally in the disapprobation bestowed upon the Censors, that I expressly stated my remarks to be applicable to the latter "alone;" adding my belief that their interference at such a juncture was condemned by the members of their own body. This belief, which I still entertain, was founded on the information of those on whom I could depend; and, for the honour of the College, I trust that the feeling is general.

It would obviously have been unfair to attribute an act of the Trustees of the Hunterian Museum, to the Censors of the College of Physicians, had not one of them thought proper to publish a full and circumstantial detail of what had passed at one of the meetings, thus claiming for himself and his colleagues the merit, and consequently entailing upon them the responsibility, of the part which they had taken in the discussions. This letter, as well as that signed "A Friend to the late Censors," appeared in the "Lancet;" a paper which few have had the boldness thus *openly* to countenance.

The writer of this anonymous letter asserts that my exculpation of the College, above-mentioned, is a "severe imputation" upon that body, and contrary to my obligation to do every thing "*in honorem Collegii*;"—that is, it is held up as a violation of my oath that I should presume to say that the Censors have acted injudiciously, or that any of their brethren think so. But, in order to make his case the stronger, he has given a quotation, with inverted commas, as my words; yet he has felt himself at liberty to alter and to strengthen the expression, converting the postulate "*we believe* to be condemned," into the simple asseveration "*is* condemned;" which, however, I regard as of no importance, farther than as it is an illustration of the spirit which pervades the whole.*

With a view to show that their conduct on this occasion was not disapproved of by the members of the College of Physicians, the "Friend to the late Censors" quotes the opinions of two individuals,—yours, Sir, and that of the senior Censor. With regard to you, we are informed that "a statement of their conduct" was deemed "perfectly satisfactory;" but, as no part of the statement is given, and of your answer, "written by the Registrar," only the two words above quoted, it is obviously impossible to know of what nature the statement was, or how far the approval extended. One thing, however, is quite clear, that a "statement" *was* deemed necessary; a circumstance at variance with the assertion of this writer, that he has heard "of no one besides Dr. Macleod who condemns them:" for I have not the vanity to suppose that my opinion would have been regarded as sufficiently important to induce the Censors of the Royal College of Physicians to draw up a "statement of their conduct."

If you, Sir, really deemed their interference with the affairs of another corporate body, at a time when they were assailed by a disaffected party among themselves, "perfectly satisfactory," I can only regret that the opinion of so eminent an individual should in this respect be opposed to that of a large proportion of the respectable part of the profession. But if it should appear (as I have *strong* reason to believe) that Dr. Macmichael

* This writer calls upon me to give him the names of those who condemn the conduct of the late Censors. Had I accused them, on the authority of others, of any immoral act, he would have had a right to make this demand; but, as the extent of my censure implied only a doubt of their absolute wisdom, it is too much to expect that I should obey such an unreasonable request. For me to do so would be to take dishonourable advantage of opinions expressed in the course of conversation: at the same time I am satisfied, from the character of the parties, that, were it of any importance for the Censors to be acquainted with their names, they would not be deterred from declaring themselves by the vapouring threat about exclusion from College offices, or from any dread of being regarded by this writer as "deficient in sense of moral obligation."

did not write *as Registrar*, and that the statement, together with the approval, had reference solely to the part which the Censors had taken with regard to the Licentiates, and to the circumstance of a Treasury minute putting it out of the power of the Trustees to admit them on the same terms as the Fellows, then it will remain for the "Friend to the late Censors" to explain on what grounds he has taken upon himself to extend your satisfaction touching one particular point into a general approval of their conduct with respect to the College of Surgeons.

But the attendance of the Censors at the Board of Trustees of the Hunterian Museum is represented by their "Friend" as a point of conscience, touching which they "swore a solemn oath," and "publicly received the Sacrament." Now, Sir, if it really was so imperative a duty, and if they have done all this for conscience sake, I cannot but think it a "severe imputation" upon you and Dr. Frampton, higher and senior officers, as well as men of more influence, to have absented yourselves from every one of these meetings, and left to the juniors the undivided honours of the whole transaction.

In the letter alluded to, it is acknowledged that the Censors were advised to send their explanation to the Medical and Physical Journal. Notwithstanding this recommendation, which sufficiently showed the channel which *you* regarded as most proper for its communication, the "Friend to the late Censors" selected the "Lancet!" a journal teeming with abuse of the College and its members; in which, accordingly, a document, but not the "statement," appeared the week after an illiberal attack upon that body, which I had absolutely refused to publish.

I fearlessly refer to the pages of the "London Medical and Physical Journal," as an index of my feelings towards the College of Physicians, and as the best proof that I have never endeavoured to create or to foster dissentions among men, whose mutual interest it is to avoid such unpleasant discussions. I beg, however, not to be misunderstood. I do not wish to exculpate myself from the charge of having censured the conduct of the late Censors, which, as a public Journalist, and touching a public question, I had a right to do; but I do wish to rebut the accusation of having thereby deviated from the consistent line of conduct which I have throughout observed towards the College of Physicians as a body, and towards the profession in general.

I have addressed this letter to you, Sir, in the present form, because I have too high a respect for you, and too much regard for my own character, to think of giving it publicity through the same medium which the "Friend to the late Censors" has had the indiscretion to adopt: and I must say that I should, indeed, regard myself as deserving the "strongest reprobation," had I presumed to offer such an insult to the College of Physicians as I conceive him to have done, by selecting, as the channel of his communication, a paper, of which it would not become me to speak in the terms which it merits. Indeed, when I consider the medium through which the letters in question have been published, I feel that some apology is necessary for noticing them at all; and I certainly should not have done so had not the circumstances under which they appeared given to them somewhat of an official form; the latter containing references to various papers, which has led to the belief that it proceeds from the late Censors themselves.

I trust that, in the foregoing remarks upon the conduct of the late Censors, which the circumstances have rendered unavoidable, I have not departed from that temperance of language and fair line of argument which Gentlemen ought to adopt towards each other: and in conclusion I beg to say, that I shall not be induced, by any consideration whatever, to prolong this discussion. It would, indeed, be endless, were the Editor of a Journal liable to be drawn into a controversy with every one from whom he differed in opinion. Both sides of the question have now been stated, and those who feel any interest about it will form their own judgment on the subject.

I have the honour to be, Sir, your most obedient humble Servant,

RODERICK MACLEOD,
Licentiate of the Royal College of Physicians,
and Editor of the "London Medical and Physical Journal."

*To* SIR HENRY HALFORD, BART.
*President of the Royal College of Physicians.*

P. S. *(Saturday, Nov. 11th.)*—I have just been informed that the anonymous letter, so frequently alluded to, has been acknowledged, in the Lancet of to-day, as having proceeded from one of the late Censors. I take this opportunity of stating that it is not my intention to read, and consequently that I cannot answer, any thing which may hereafter appear in that publication. R. M.

---

*Remarks.*

The above documents sufficiently explain the feelings, and set forth the arguments of the two parties. It would ill befit us to interfere, or give any opinion on the question, when every reader has the same data to ground his opinion on that we have.

In respect to the observations which we made on this subject, in our last number, we have but little to add. We distinctly stated that, "*we knew not how, where, or why this invidious prohibition started into existence,*" but that we had no hesitation in designating it a foul blot on the medical annals of England. We withdraw not one iota of the above sentiment. It is now clearly proved that the late Censors had no hand in originating this regulation respecting the admission of licentiates, and therefore they are absolved from all blame. That it was a *dormant* regulation, we appeal to the fellows and licentiates themselves. Did the *latter* require personal introductions from the *former?* We never heard of such a thing; and we can attest that we were never questioned as to our claim to admission, on proper days, in the character of licentiate. It is clear then that the treasury minute was a dead-letter till the late proceedings, when it was revived, unavoidably no doubt, with the other parts of the treasury minute which had not been complied with by the Curators. For this we do not blame the late censors, and are bound to believe that they did their best to rescind the regulation entirely.

But it becomes necessary to notice the letter of SOCIUS, in the November Medical and Physical Journal. This gentleman alludes to the "unkind feelings" which are alledged by us as existing in the minds of the Licentiates towards the Fellows of the College. We never made any such assertion. We said that the marked degradation which meets the eye of the Licentiate, would be likely to transmute into open hostility what now exists in the shape of smothered indignation. This indignation arises from the state of *degradation,* (we repeat the word) in which the Licentiate is placed by the anomaly of his situation, without any reflection on the Fellows of the College, who cannot alter the existing state of things. We ask SOCIUS if the document produced by Dr. Ager does not prove the degradation of the Licentiate. The very Government itself degrades him, by either not recognizing such a being in the profession, or excluding him from a privilege ceded to every member of the College of Surgeons. If SOCIUS will attempt to persuade the Licentiates that they have no reason in the world to be discontented with their lot, he knows little of human nature. Can a Blane, a Babington, a Philip, an Armstrong, and fifty others of this metropolis, proceed to the house of Socius, cap in hand, to ask *permission* to visit the Hunterian Museum without feelings of smothered indignation? If they can, they are not men.

We have, on several occasions, exhorted every father in the British dominions, who has a son destined to practise as physician in England, and especially in the metropolis, to give him his classical and scientific education at Oxford or Cambridge, and his medical education in London, Dublin, Edinburgh, or Paris, as he may think fit. By this plan his son will become a Fellow of the College of Physicians, and evade the degradation of the Licentiate. This plan is also, in our opinion, infinitely preferable to any other, in every point of view. We *know* that our observations on this subject, on a former occasion, have produced a strong impression on the minds of those who are bringing up their sons as physicians.

But as the change which may be effected thus, in the proportion between Fellows and Licentiates, must be very slow, and can never entirely cure the invidious distinction which now exists, we think that an act of liberality on the part of the College, which would be perfectly compatible with the safety and stability of its charter, and eminently calculated to disarm the medico-radicals, while it cemented the bond of union among all the more distinguished physicians of the kingdom, is highly worthy of attention from the present enlightened president of the College. We mean an annual admission of a certain number of physicians (of other Universities than Oxford or Cambridge) who may have acquired the esteem of the public and the profession at large, by their probity, talents, learning, and medical science. Such a prospect or premium for merit would prove an unceasing stimulus to the whole body of physicians, while the annual influx of talent into the fellowship would soon render it the most distinguished College in Europe. No change, in our humble opinion, should be made in the path which now leads to the *right* of a fellowship; but the *grace* of admission should be liberal, and accorded *only* to merit. The parsimonious exercise of this prerogative, once in three or four years, towards an individual, is really calculated to engender jealousy and discontent, instead of emulation, hope, and concord, between the two classes of physicians.

---

### IV.

### PATHOLOGY AND TREATMENT OF CROUP.

*Spreull's Court, Glasgow, 5th Oct.* 1826.

Sir,—In reply to some remarks which you have introduced into your Journal for Oct. 1826, page 436, I beg leave to say, that I had neither seen nor heard of M. Guersent's Articles on Croup, nor of Dr. Bretonneau's Memoirs on that subject, when I published my remarks in the Edinburgh Journal for April, 1825. I have also reason to believe, that these authors and their opinions were quite unknown to those practitioners, who, in this town, first introduced the practice of applying lunar caustic in what was called *Sore Throat ending in Croup.*

As for the pathological fact, that the fibrinous effusion in croup, at least in some cases, commences on the tonsils, and thence spreads to the velum, pharynx, larynx, windpipe, and œsophagus, this was so strikingly the case in more than one fatal case which occurred under my own care, that I could not omit to observe it. The coincidence in pathology between Dr. Bretonneau and myself, has been purely the result of two individuals attending to the same disease, without any knowledge of the labours of each other; and of course it confirms the observations of both.

The coincidence in practice between Dr. Bretonneau and the practitioners in this town, who, some 7 years ago, tried lunar caustic in the *sore throat*, as it was styled, which so frequently *ended in croup*, is, I believe, as purely accidental; and also highly confirmative of the utility of this application of escharotic remedies. It was to these gentlemen I referred in my paper, when I stated that I had received this practice from others, in whose hands, as well as in my own, it had proved successful.

The following is the progress of my knowledge of the subject.

In 1812 or 1813, I had occasion to see two cases of what was called *Sore Throat ending in Croup*. They were under the care of the late Mr. Stenhouse of this town, and in one of them I repeatedly applied, under his direction, a strong infusion of capsicum to the tonsils, by means of a bit of rag mounted on a pencil. Both cases terminated fatally; and of course indelibly impressed my mind with the alledged fact, that *putrid sore throat* was liable to *end in croup*.

In 1819, the same disease prevailed very much in this place, and I had an opportunity of inspecting the bodies of several children who died of it.

In May, 1820, Dr. Thomas Brown read a valuable paper on this disease, in the Glasgow Medical Society, and related, amongst other cases, one in which the application of solid lunar caustic appeared to be successful in preventing the extension of this disease to the larynx. I afterwards understood that Mr. James Watson had found a solution of the same substance successful in one or more cases.

In 1821, this disease proved fatal to two children under my own care. In both, the fetor of the breath and the sloughy appearance of the effused lymph, were remarkable. In the first case, indeed, I was surprised, on dissection, to find the tonsils and uvula entire, and coated over only with an effusion; for I had laid my account to find a gangrenous loss of substance in these parts. My opinions regarding this *Sore Throat ending in Croup*, had till this period been wavering; but I now announced to several of my medical brethren, that what had been considered ulcers and sloughs in this disease were nothing else than effused lymph, the progress of which over the velum and uvula, and towards the alimentary and respiratory passages, I had distinctly observed.

In the 2d of these two cases, I called in Mr. James Watson, who applied the solution of lunar caustic, but without success. In two subsequent cases, however, (one of which was also seen by Mr. W. along with me,) having found the solution effectual in procuring the detachment of the effused lymph, and apparently in preventing the extension of the disease to the larynx, I judged that I ought not to allow a remedy, which promised to be useful in so terrible a disease, to remain unknown; and accordingly I published my remarks in the Edinburgh Journal. Whatever motives others might have to let their observations pass *sub silentio*, I had none; and, therefore, published what I thought, and what I had seen of this disease and of this mode of treatment, taking credit, no doubt, to myself for what belonged to me regarding the pathology, but ascribing to others the invention of the escharotic practice, without touching on the delicate question of priority. Notwithstanding this, a sort of an attack was made upon me in the Glasgow Medical Society, not by Dr. Brown nor

Mr. Watson, but by a third person, as if I had wished to appropriate other men's discoveries to myself. Notice of this attack was taken in a late Number of Anderson's Journal; it was there styled a reclamation.

It gives me sincere pleasure to think, that in consequence of your review of Dr. Bretonneau's Work, the pathology of this disease is likely to be more generally understood, and that a fair trial will be given to the escharotic treatment. Whether muriatic acid or argentum nitratum answers best, must be determined by experience only; but the facts that CROUP BEGINS ON THE TONSILS, and that BY ESCHAROTIC APPLICATIONS IT MAY BE PREVENTED FROM EXTENDING FARTHER, are of the highest importance, and cannot now fail to attract the consideration of your numerous readers, and of the profession in general.

I am, Sir,
Your most obedient Servant,
W. MACKENZIE.

---

### V.

### QUALIFICATIONS OF ARMY MEDICAL OFFICERS.

### *Hospital Assistant.*

The Candidate for this, the first appointment in the Medical Department, must be unmarried; not under twenty-one, nor above twenty-six years of age; he either must produce Certificates of having served a regular Apprenticeship, of not less than three years, (to a Member of the Royal Colleges of Surgeons, of London, Dublin, or Edinburgh, will be preferred) and of attendance for one year at least, in an Hospital or Infirmary of celebrity; or if he has not served an Apprenticeship, he must produce Certificates of two years' attendance in an Hospital, with one on practical Pharmacy. He is to possess a Diploma from one of the Royal Colleges mentioned, and must exhibit Certificates of regular attendance on courses of study on the following branches of professional knowledge, at established schools of eminence: viz:

| | |
|---|---|
| Anatomy ........................ | 18 Months. |
| Practical Anatomy .................. | 12 do. |
| Chemistry and Practical Chemistry .. | 6 do. |
| Materia Medica ..................... | 3 do. |
| Botany .......................... | 3 do. |
| Surgery ......................... | 6 do. |
| Theory of Medicine and Physiology.. | 6 do. |
| Practice of Medicine .............. | 6 do. |

and of Clinical Lectures on the Practice of Medicine and Surgery.

It will be considered an additional recommendation to Gentlemen entering the Service, to have attended Lectures on

Diseases of the Eye, and on
Forensic Medicine, and likewise Public Establishments for the treatment of Diseases of the Eye, and of Mental Derangement.

Gentlemen who have had an University Education will always be preferred. It is desirable that all should have studied Mathematics, Natural Philosophy, and Natural History; but a liberal Education, and a competent knowledge of the Greek and Latin languages, are indispensably requisite in every Candidate.

*Assistant Surgeons and Surgeons.*

With the above recited Qualifications, Hospital Assistants will be entitled to promotion as Assistant Surgeons and Regimental Surgeons; but every Gentleman must have served five years at least in the junior appointments, before he can be promoted to the rank of Regimental Surgeon. In addition to competent professional knowledge, the greater the attainments of Gentlemen in various branches of Science, the more eligible will they be deemed for promotion.

Gentlemen already in the Service, are earnestly recommended to avail themselves of every opportunity of adding to their knowledge, by attending Universities or Schools; for which purpose, every facility in his power will be afforded by the Director General. They are especially desired to transmit to this Office Statements of such Classes as they attend subsequently to their Examinations before this Board, either on professional or on other branches of Science, that the same may be duly registered here: and every Officer must be prepared to undergo further Examination before he can obtain promotion, or return to the Duties of the Service from the Half Pay List.

*Physician and Surgeon to the Forces.*

Medical Officers are encouraged to look forward to the appointment of Surgeon to the Forces, and of Physician to the Forces; and to endeavour especially to qualify themselves for either rank, according to the bent of their inclinations and to their previous studies.

It is expected that the Candidates for the Commission of Surgeon to the Forces, shall have attended a Public Hospital of celebrity at least two years; and it is desirable that one of them should have been passed at a London Hospital.

The rank of Physician to the Forces, requires in addition to the knowledge and experience to be gained in the regular progress of study and of Service, that the Candidate should be a Fellow or Licentiate of the Royal College of Physicians of London, or a Graduate of the Universities of Oxford, Cambridge, Dublin, Edinburgh, Glasgow, or, under the Regulations lately adopted by them, of the Universities of St. Andrew's, or Aberdeen.

Although the British Schools are specified, it is to be understood that Candidates who have received regular Education in approved Foreign Universities, or Schools, will be admitted to Examination.

In all the steps of promotion in the Medical Service of the Army, they who give the best proofs of diligent exertion in the performance of public duty, and of attention in the acquirement of practical knowledge, by taking every opportunity of adding thereto, will be regarded as the most eligible for advancement.

*Army Medical Department.*

*Note.* All Letters to be enclosed, *unsealed,* to "The Right Honorable the Secretary at War."

### VI.

*To the Editor of the Medico-Chirurgical Review.*

Sir,—The name of Cuvier, and the well earned honours of Professor Dumeril ought, one would suppose, to protect these great men from being implicated by a nameless reviewer, in a physiological misrepresentation with so humble an individual as myself. They are, however, accused, in the last Number of "the Edinburgh Journal of Medical Science,"* of abetting a theory built by "we" himself, and founded upon what he terms "*the sugescent power of 'the heart'—the suction of the lungs, &c.*"

This theory, the critic condenses into seven propositions, and gives them to *his readers* as the deductions which I have drawn from my experiments. *Your readers* will no doubt, be somewhat surprised when I assure them, that in six of these propositions I cannot recognize a single phrase as my own, nor one *pure* element of any of the deductions which I have made from my experiments.

My book is charged with asserting, that atmospheric pressure is the *sole cause* of the motion of the blood of the veins towards the heart, and the illustrious philosophers just mentioned, are charged with abetting this assertion. The critic manifestly mistakes their application of the word "uniquement," as completely as he has misapplied the loose calculation translated from Haller, by transferring it from the black blood of the pulmonary *orbices*, to the red blood of the pulmonary veins. This vague approximation, given by Haller, to show how little the pressure of the air can *impede* the current in the pulmonary *arteries*, is not corrected by our critic, by applying to it any of the lights which modern science and discovery have shed upon the problem to which it refers. Yet he very modestly terms it "*infallible*," and appends to his translation, Q. E. D!!!

The names of Cuvier, Dumeril, and Laennec, men, whose contributions to science are destined to adorn the brightest pages of our intellectual history, should be respected. As to myself, the following short extract, will show how little "the elegant rotundity of my deductions," *squares* with the reviewer's six propositions. (The 7th relates to the cupping-glass.)

"From what we have seen in the memoir, and from what has been said in the supplement, it is evident, that in the quiescent living animal, the only demonstrable active powers employed by Nature to propel the contents of the veins towards the heart are,

"1st, The impulse given by the pressure of *the heart itself* continued *through* and propagated by the arteries. By this power the blood is sent into the situations where it can be most favorably acted upon by,

"2ndly,—Atmospheric pressure, diminished, or entirely taken off around the cardiac ends of the venous tubes, but left entire around every other part of their surface.

"3rdly,—Gravitation, &c."†

Had the misapprehensions of the northern critic been confined to myself, I should not have troubled you, nor the public with any notice of them. But when a man appears to build up a theory for the author whom he is reviewing; and when he gratuitously implicates in that absurd theory, three of the most profound philosophers of the age, merely to overwhelm them in the common ruin, by "*we*" and "our infallible calculation;" it would be allowing a stigma to remain on the literary integrity of the country, not to afford the learned reviewer an opportunity of making the "*amende honorable*." I am Sir, your obedient Servant.

D. BARRY.

*7, Great Mary-la-bonne Street, Cavendish Square,*

*10th December*, 1826.

---

* Page 462.

† Experimental Researches, (p. 56 and 57.)

### VII.

### GLASGOW REGULATIONS.

*Regulations respecting Degrees in Medicine and Surgery, in the University of Glasgow, as fixed by the Senate, 17th April, 1826.*

MEDICINE.

I. Every Candidate for a Medical Degree, must bring evidence that he has reached the age of twenty-one.

II. He must bring evidence of having attended for *four* years, some University, in which Medicine is regularly taught, or the Lectures delivered in the Theatre of the College of Surgeons, Dublin; or those delivered in London: one of these years at least, he must have attended the University of Glasgow.

III. He must produce Certificates of having attended the following Medical Classes, in the above-mentioned Schools—each Course being reckoned to last during *six* calendar months, viz. Anatomy during two such courses: Chemistry, two courses: Institutions of Medicine, one course: Materia Medica, one course: Midwifery, one course: Surgery, one course: Botany, one course in a University Hospital, during twelve months. *N. B.* Two London courses of between three and four months each, to be reckoned equivalent to one six months' course.

IV. Each Candidate for a Medical Degree, must announce his intention, and lodge the requisite Testimonials with the youngest Member of the Medical Faculty in the University, two months before the time of Graduation; that is, by the 1st of March, and the 1st of June, otherwise he cannot be taken on Trial till the following year.

V. Every Candidate for a Medical Degree, shall undergo three examinations: the first, on Anatomy and Physiology; the second on Chemistry and Pharmacy; and the third, on the Practice of Medicine. He shall likewise write a Latin Commentary on an Aphorism of Hippocrates, and a Medical Case.

VI. The Degrees shall be conferred on those Candidates who have acquitted themselves to the satisfaction of the Examinators, on the last Wednesday of April, and the first Wednesday of August, in each year, and at no other time.

SURGERY.

The Curriculum of Students who mean to take Degrees in Surgery, to be three years; during which period, they must attend the following courses of *six* months each, or the equivalent London courses as specified in the case of Physicians; viz. Anatomy, two courses; Surgery, two courses; Chemistry, one course; Institutions of Medicine, one course; Practice of Medicine, one course; Midwifery, one course; Materia Medica, one course; Hospital, during twelve months. One of the years of attendance, at least, must be in the University of Glasgow: the Degrees in Surgery to be conferred, only on the last Wednesday of April.

*** The Fee to the Library, for the Degree of M.D. is £15.—Duty on the Stamp, £10. 3*s*.—that for C. M.; or Chirurgæ Magister is £10. 10*s*.

### POST-MORTEM CHANGES.*

[Veterinary School of Alfort.]

As dissection rarely takes place till many hours after the death of the human subject, there must doubtless take place, in that interval, many changes, which are usually attributed to previous disease. On this account it is necessary to make experiments on animals, in order to ascertain what these changes are.

Monsieur Rigot, as chief anatomist at the Veterinary School of Alfort, had the means of prosecuting this interesting investigation, in which he was assisted by Dr. Trousseau, formerly an interne of the General Hospital of Tours.

In the study of pathology, it is necessary to know what are the appearances of organs and tissues in the sound and living state, else we cannot appreciate the alterations produced by disease. The next step is to ascertain what are the immediate changes induced by the cessation of life anterior to the commencement of the putrefactive process.

In the Veterinary School of Alfort, a certain number of horses are given to the students every week for the purpose of experiments and operations. These animals are afterwards killed and dissected. In many of the examinations which our authors made, the hearts of the animals had not ceased to beat, when the dissection commenced—consequently they had the means of ascertaining the appearances of the parts examined before any *post mortem* alteration could obtain. A number of horses, to all appearance sound in health, though lame or otherwise rendered useless, were killed for the purposes in question. Examinations were made at various intervals of time from the extinction of life, and the changes carefully noted, together with all other attendant circumstances that might be likely to influence these post mortem changes. Many experiments were also made on dogs; but in these animals the appearances of parts do not accord with those in man, so well as the appearances in the horse—especially as regards the intestinal tube. The present paper is limited to the changes produced by death in sound parts. In a future memoir, our authors mean to furnish us with the changes induced in inflamed structures, by the same event.

1. *The Heart and Vessels.* Twenty eight horses were examined instantaneously after death. The lining membrane of the heart was found to be thin, white, transparent, mottled in some points. That of the arteries was of a yellowish white—and that of the veins was of a very clear white appearance. Two exceptions only occurred, where yellowish copper-coloured spots were observed on the internal surface of the aorta, and for which no satisfactory explanation could be offered, unless they were indications of incipient disease. These spots did not penetrate beneath the lining membrane of the artery, the coats being neither thickened, rugous, nor softened. Such a number of dissections, our author thinks, is quite sufficient to settle the question as to the normal condition of the internal surface of the blood-vessels, in life and health. But to clear all doubt, some *vivisections* were performed to ascertain whether any change could take place during the very act of dying, and before the parts could be exposed to view.

---

* Recherches Necroscopiques sur quelques Alterations, que subissent, après la Mort, les Vaisseaux Sanguins, les Poumons, et la Membrane Muqueuse Gastro-pulmonaires à l'Etat sain. Par M. M. Rigot et Trousseau: Archives, Octobre, 1826.

We shall now take a rapid glance at some of the experiments, in order to shew the successive appearances at different periods *post mortem.*

*Exp.* 1. A dog, two years old, was strangled, and examined *half an hour* afterwards. No water in the pericardium—internal membrane of the heart not the least tinged with blood. The same of the arteries and veins.

*Exp.* 2. A horse, lame, but otherwise in good health, was pithed, and opened *one hour* after death. There were six ounces of fluid in the pericardium. The lining membranes of the heart, veins, and arteries were entirely free from the least tint, although there was blood in these vessels. In some of the blood from this animal a piece of aorta was immersed. In 24 hours it was as red as scarlet, and this tinge could not be removed by repeated washings.

*Exp.* 3. A horse was opened *two hours and a half* after being pithed. The result was the same as in the last experiment. In the next experiment, *five hours* were suffered to elapse, and still there was nothing particular observable in the vessels or heart.

*Exp.* 4. In a dog, opened *ten hours* after death, the auriculo-ventricular valves were found to be of a deep red colour. The internal surface of the cavæ was also of a dark brown colour, which could not be washed off.

*Exp.* 5. A horse was dissected 14 *hours* after death. The whole of the aorta presented a red colour, as did the pulmonary artery. Some portions of the venæ cavæ were tinged, others not.

*Exp.* 6. After *sixteen hours*, a horse was carefully examined. The auriculo-ventricular valves were of a scarlet hue. The right chambers of the heart, which contained much blood, were deeply tinged—and the aorta was like scarlet.

We shall pass over the other experiments, as only multiplying the foregoing appearances. Our authors next endeavour to account for the causes which favour or retard this imbibition of the vessels. He says that, the more plastic the blood is—the less serum it contains—the less it parts with its colouring principle, so much the less it colours the tissues with which it is in contact. It sometimes happens that the chambers of the heart and also the vessels contain clots of fibrine, and at the same time some cruor: in these cases there will be corresponding patches of red, and other portions untinged. In general, the vessels containing venous blood were more tinged than those containing arterial blood. The position of the dead body has great effect in producing or preventing this tincture of the vessels. The more blood, the more the tinge of its containing tubes. If an organ, as the lungs, be in a state of congestion or inflammation before death, as soon as the vital laws have given way to the laws of decomposition, the blood begins to tinge the containing vessels. It is thus that, in cases of pulmonary inflammation, the pulmonary vessels are often found red. The nature of diseases has also an influence on this coloration of vessels. Our authors have examined several cases of what are called highly inflammatory fevers, such as are characterised by hard pulse, great heat, redness of the face, exaltation of the intellectual functions, &c. but never could discover the least trace of redness in the vessels. In fact, they consider the state of the blood, in such cases, to be unfavourable to the production of the phenomena in question. On the other hand, in those who die of erysipelas, large and deep-seated abscesses, small-pox, scarlatina, typhus, &c. the coloration of the vessels is a common occurrence. In these, the blood is in a dissolved condition—blacker than usual—and disposed to give out its colouring matter to the containing vessels. Our authors warn the physician against applying too rigorously these experiments on animals to the pathology of man. The bodies of horses putrefy much sooner than those of the human race—and the blood of herbivorous animals is more disposed to produce this imbibition than the blood of omnivorous creatures.

Many speculations have been entertained by pathological writers on this phenomenon in the blood-vessels. Corvisart very often met with this redness of the aorta, and never could satisfactorily account for it, as it was generally unaccompanied by any thickening of the tinged tunic. Dr. Frank, of Vienna, alludes to this coloration of the arteries, but gives no details by which one can judge of the condition of the subject or attending circumstances of the case. Mr. Hodgson describes this red appearance of the arteries, but does not consider it as dependent on acute inflammation. It was not accompanied, in his observations, by thickening of the tunic, or lymphatic effusion between the coats. In fact, he is at a loss whether to attribute the phenomenon to a morbid action or to a post mortem change. M. Laennec goes farther than Mr. Hodgson ; for he says :—that the redness of the lining membrane of the heart and large vessels should, in no case, and however deep the tinge, be considered *alone*, as proof of inflammation. He looks upon it as a purely cadaveric change, and more especially owing to a long struggle or agony before death. Nevertheless some able pathologists of the present day in France, range themselves on the opposite side, and bring forward facts in support of their conclusions. The principal pathologists of this class are, Messrs. Bertin, Bouillaud, and the younger Andral. In respect to some cases published by the latter, with the view of supporting his doctrine, our authors think they prove just the contrary of what they were intended to prove. Thus, a case is detailed of pulmonic inflammation, where the dissection was made *thirty-six hours* after death, and where the lining membrane of the aorta was found red. In another case, *thirty-five hours* intervened between death and dissection, and blood was found in the cavities of the heart. Here the lining membrane of these cavities was also found red. Our authors are of opinion, after the experiments which have been detailed, that these appearances afford no certain proof of inflammation, and that they were *post mortem* changes.

Finally, our authors consider themselves entitled to conclude—1mo, That redness is no certain sign of inflammation in the lining membrane of the blood-vessels and heart. 2dly, That, hitherto we have no criterion for distinguishing inflammatory redness from *post mortem* coloration. 3tio, That the redness of the vessels is so much the more conspicuous, in proportion as the blood is more fluid and more darkly coloured—in proportion as the surrounding tissues are naturally or accidentally gorged with blood—and in proportion to the length of time that has elapsed since death. 4thly, That although these conclusions should not be very rigidly applied to the human body, yet, allowing for the obvious differences between man and animals, considerable analogy will be found to exist, and much may be learnt from these experiments on the latter class of beings.

The results of their experiments respecting the appearances of the alimentary canal, are reserved for another paper.

### POISONING BY BITTER ALMONDS.

Mr. Kennedy, of Virginia Terrace, met with a case of this kind in Sept. 1825. He was called to a man who had been observed to fall down, apparently while making water against some wood in Trinity Street, Borough. He was pulseless and breathless, and no blood, or very little, could be got from the jugular vein or arm. Some brandy was poured down his throat, and then a little blood flowed; but life was extinct. The mouth emitted a strong flavour of hydrocyanic acid, and a quantity of bitter almonds was found in the man's pockets.

On dissection, the vessels of the brain were found gorged with blood, as in apoplexy, but the substance of the brain healthy. The stomach was found immensely distended, and many pieces of the bitter almond among the ingesta. The contents of this organ had a strong flavour of Prussic acid. The eyes were bright and glistening, and, 24 hours after death, there was some warmth about the chest. There was no inflammation in any organ of the body.

*Remarks.* Some doubt may exist as to the cause of death in this case. The vessels of the brain being distended, as in apoplexy, it is possible that a sudden congestion in the vessels of that organ, occasioned or accelerated by the indigestible mass and the great distention of the stomach, might have led to the fatal event. On the other hand, the nature of the substance found in the stomach and pockets, the flavour of Prussic acid in the mouth and stomach, the glistening eye, and the long retention of heat about the chest, are strong evidences of poisoning by hydrocyanic acid.

### TOBACCO IN TETANUS.*

In a former number of this Journal, we gave some account of Dr. Anderson's experience of tobacco in tetanus, as published in the first volume of the Edinburgh Medico-Chirurgical Transactions. The present contribution is a sequel to Dr. A.'s previous paper.

Several opportunities have been afforded our author by his professional brethren in Trinidad, of administering tobacco in idiopathic tetanus, and this medicine has proved successful in his hands. Only two cases additional are here related, the first of which terminated fatally. The second was a negro boy, 12 years of age, to whom our author was called by Dr. O'Connor. He had unequivocal opisthotonic spasms, recurring every three minutes—the convulsed and distressed countenance distinctive of tetanus—perspiration on the surface—quick pulse—furred tongue—trismus—constipated bowels—thirst, &c. The tobacco baths, strongly impregnated, were advised till nausea, and even vomiting should ensue. Purgative. The tetanus resulted from a punctured wound. Sensible effects were produced by the bath, and the spasms were controlled. Great difficulty, as usual, occurred in opening the bowels, which required calomel and gamboge. These brought away unnatural motions, and some lumbrici. After several days' perseverance in the tobacco baths, the tetanus was finally overcome, and the boy recovered. "As a general rule," says our author, "four pounds of the dried plant may be boiled for an hour in eight gallons of water, and used to impregnate the tepid water of a bath."

* Dr. Thomas Anderson. Ed. Transactions, vol. 2.

### FRACTURE OF THE NECK OF THE THIGH BONE.*

Sabatier, it is well known, treated this kind of fracture by position of the limb, without any bandagings whatever: his method, subsequently modified, perhaps improved, by Mursinna, is now preferred by Dupuytren, in whose practice it is found agreeable to the patient, and less subject than others to accidents. A man who had been several years cured, by this method, of fracture of the neck of his thigh bone, walked frequently from Mendon to Paris, for the purpose of consulting Sabatier on another disease: this man had some, but very little, lameness or deformity: he died in the Hôtel Dieu from cancer on the face. Dr. Ribes, on dissecting the fractured thigh, found the cellular texture around the joint much condensed, and the tendons of all its muscles thicker than those of the other side: the capsule was greatly thickened, and nearly cartilaginous in consistence. On the articulation being laid open by a circular incision, the neck was discovered to have been broken near the head of the bone: the portion of the neck thus separated from the head was rounded upon the fractured joint, and covered with true "diarthrodial" cartilage: and this rounded portion itself was hollowed into a deep cavity in the middle of the part corresponding to the head of the thigh-bone,' and covered with "accidental diarthrodial" cartilage. The head of this bone was so singularly deformed, that it presented the appearance of a skull-cap placed between the cotyloid cavity and the new head formed in the fractured neck: superiorly, a strip of periosteum enveloped the neck of the thigh-bone, and, being attached to the edges, the caplike cavity united the upper and lower parts of the bone. The "inter-articular" ligament was sound, but shorter and wider than is natural: it connected the cotyloid and cap-like cavities; and had become so very thick as to form a "double articulation accidentally developed."

### FRACTURE OF THE KNEE-PAN.†

Sabatier also treated fractures of this bone by position exclusively: he placed the leg and thigh, perfectly extended, on an inclined plane, with cushions of oat-chaff, the foot being seven or eight inches higher than the groin: he subdued particular symptoms with bleeding, poultices, and fomentations. For seven days, at first, the pieces of bone remained more or less separated; but, by the end of a fortnight, the space between them visibly diminished, and was ultimately filled with a substance which daily grew firmer: in two months, a patient could walk; and soon afterwards had the use of his limb completely restored. Dr. Ribes dissected several knees in which the patella had been fractured many years before, and the cure accomplished by position entirely. In some the pieces were united by a thick, firm, ligamentary substance; in a few, this substance was six lines thick; in others, it constituted a simple layer between two of the pieces of bone: in one subject, the knee-pan was broken more than ten years before, and the bone, after the treatment by position, had become perfectly consolidated.

* Mémoires de la Société Médicale d'Emulation de Paris, tome ix. p. 95. Paris, 1826.

† Memoires de la Société Médicale d'Emulation de Paris, tome ix. p. 100. Paris, 1826.

### RETROSPECTIVE REVIEW

OF THE

### PERIODICAL PRESS

IN

### GREAT BRITAIN,

SINCE ITS COMMENCEMENT IN 1730.

Forsan et hæc olim meminisse juvabit.

(No. 1.)

It is now nearly a century since the periodical press (we mean the medical) first commenced its career in these islands. In that comparatively short space of time, many a work put forth the buds of hope—flourished for a time—and then, like all earthly things, decayed and vanished from the scene! As not one in five hundred—perhaps not one in a thousand, of the profession either possess or can have access to this voluminous series of essays and observations, it may not be unprofitable to dedicate a few pages of our Journal, occasionally, to a retrospective glance at some of those works, which, in their day, attracted the notice and occupied the attention of those who have gone before us. Most of the resuscitated *facts* which we shall notice will be new to the generality of readers—and some of the *reminiscences* may perchance give origin to useful reflections even among the erudite.

"Men must be taught as if ye taught them not,
"And things unknown proposed as things forgot."

It is, in truth, but a very small proportion of the "working classes," or, to use a still more significant term, the "operatives" of medical society, who have either leisure or inclination to dive into the records of past times, for the purpose of gleaning *useful* information from the vast mass of error and absurdity under which it lies buried. But we have plenty of literary miners who dig up from the bowels of antiquity, "such stuff as dreams are made on," and which produces no other earthly good than that of increasing the consumption of oil and lamp-black—employing some additional printer's devils—and augmenting the revenue by the stamp duty on advertisements. We could adduce numerous illustrations of this remark, even from the publications of 1826—illustrations which would cast in the shade the valuable specimens of medicinal lore extracted from the ancients by a Moseley, such as that—"making water through a birch broom will cure impotency"—or that "puppies, frogs, and ducks, applied to the abdomen will cure dysentery." *Moseley on Tropical Climates, pp.* 288—315.

By these observations we do not mean to discountenance the study of the ancients—far from it—but to repress the practice of inundating modern writings with the absurdities of our forefathers—a practice which, we are convinced, is too often founded on pedantry and ostentation—exhibiting unquestionable proofs that the extent of a man's reading is often in an inverse ratio to his improvement. It will be our object, in the following series of reminiscences, to keep *utility* constantly in view, and rather to disclose the merits, than draw forth the frailties of the dead. We have selected the present path of retrospection for various reasons; and should our labours be encouraged or approved, we mean, in another series of reminiscences, to exhibit some of the beauties of the ancients, from the Father of Physic down to the extinction of literature in the dark ages.

It may not be uninteresting to learn what were the reasons which induced our first periodical writers in medicine, in these Isles, to commence a line of publication which has since attained such a gigantic extent of circulation and influence.

1. "The Medical Essays and Observations, by a Society in Edinburgh," had the honour of being the first periodical work in medicine, which Great Britain ever produced, and appears (for we have only the second edition) to have commenced about the year 1730 or 31. The reasons adduced for this undertaking, were precisely those which have been adduced within a year, for a similar work, as we shall now shew.

1730. "No complaint is more general among those who apply to the study of any liberal science, than their being under a necessity of perusing such numbers of publications as are wrote on the several parts of each of them—a labour that can have no end, since one book serves only as an introduction to another, while a few pages might contain all that is new or valuable in *most of them*."—*Med. Essays, Preface.*

1826. "It is admitted that, of late years, the advance of medical knowledge has been unprecedented, and that publications and journals, in keeping pace with this improvement, have increased to an almost incalculable extent. The annual purchase of them has thus become a heavy tax. Nor is this the only inconvenience experienced. The practitioner, in attempting to avail himself of the various new researches which are daily published, is too frequently compelled to consume much valuable time in separating what it is important for him to know, from diffuse and often trivial detail."—*Edinb. Journal of Medical Science, No.* 1, *Jan.* 1826.

It would be difficult to find two passages more similar, or rather more identical than the above, with a century between them, in the whole range of medical literature. The elder journalist goes on deploring the multiplicity of books, and the badness of their quality—"for though the number and sizes of books are very great, as evidently appears on viewing the catalogues of the scripta medica, yet how few of them are in esteem?" And he moreover broadly hints that the greater number of medical publications, in his day, were written "for any thing rather than with a view to promote the science of medicine"—so that the modern reader has the consolation of knowing that his forefathers were just as discontented as himself—and with far greater reason. For the journalist, a century ago, proposes a periodical work as a remedy for the increasing evil—and now we have no less than eight medical journals, and nearly an equal number of periodical medical transactions, within the British Isles! How would Munro *primus* (who, we suspect, was the editor of the work before us) stare, if he could burst his marble cerements, and view the numerous progeny, of all shapes and sizes, which have descended from his original stock! Some future journalist will, perchance, smile at the narrow bounds within which periodical medicine is even now confined. A time, it is possible, may come, when a medical journal will issue with each clock that strikes, from nine in the morning till ten at night.

The Edinburgh Essays lived for the space of about five years, and are contained in six octavo volumes—the principal personages who figured on the stage during that period, were Dr. Munro, Dr. Plummer, Dr. Porterfield, Dr. Martine, Dr. Paton, Mr. Douglas, Dr. Drummond, Dr. Innes, Dr. Barry, (of Cork,) Dr. John Armstrong, Dr. St. Clair, Dr. Louis, Mr. Jos. Gibson, Dr. Pringle, Dr. John Fothergill, Dr. Simson, Dr. Gilchrist, Dr. Short, Dr. Crawford, Dr. Whytt, &c. &c. These were the main contributors, and many of them co-editors of the work—and yet it fell in less than half a dozen of years, and is now only to be found in collections of old books. We have taken the pains to examine these six volumes, and are sorry to observe that we are not able to enrich this article with more than one or two facts out of the collected labours of so many eminent men. One of these facts relates to a subject which has lately occupied the attention of obstetrical practitioners—namely, the complication of ascites with pregnancy, and the propriety of paracentesis under such circumstances. The case which we are about to notice appears to us to have been an hydatid in the abdomen, probably attached to the liver, which burst and produced ascites, during utero-gestation.

*Case. By Mr. Laurie, of Selkirk.* A woman, 36 years of age, who had borne six children, perceived in the month of November, 1739, a tumour under the false ribs, which stretched itself gradually downwards till, passing the umbilicus, it filled the upper part of the abdomen to the size of nine months' pregnancy. On the 12th of August, 1740, she thought she felt something crack within her, when the tumour suddenly became diffused through all parts of the abdomen, being previously more circumscribed about the upper parts of that region. She immediately became sick, faint, and ultimately feverish. Various medicines were used, but to no effect. Dr. Plummer being now in that part of the country, advised paracentesis, which was consented to, and on the 13th September eight Scotch pints of water were drawn off. In a few days the belly began again to swell, and on the 22d September four Scotch pints were evacuated. On the 2d October it was necessary to operate again, and five pints were discharged. It was now discovered that the woman was about five months gone in pregnancy, and from this period every thing went on well, and she was safely delivered of a living child. She had no return of the dropsy. We have now placed on record a considerable number of cases where this embarrassing complication existed, and where the operation of paracentesis was found both safe and salutary.

We shall notice but one other fact, or rather observation, contained in these volumes. Dr. Drummond, president of the College of Physicians in Edinburgh, in a very judicious Essay "on the Improvement of Medicine," (vol. i. p. 271) clearly anticipates some modern authors who have so strongly advocated the inflammatory nature of certain dropsies, and the propriety of blood-letting in the same.

In the last volume of the Medical Essays, the editors, as has been often the case since, die in the most flourishing condition imaginable. "The demand for our collections at home, and the translations of them in different parts of Europe make us *flatter* ourselves," &c. But still they would not let *well* alone—they must do *better*. A society was formed on an extensive scale, in which all the branches of natural knowledge were to be improved, as well as all the branches of medicine. With this accession of strength they commenced a new and brilliant æra, with two secretaries of state, one of whom was no less a personage than the celebrated Plummer. But, alas! how vain are human hopes and wishes! The rebellion of 1745 broke out, and it was nearly ten years before the new society could bring out their first volume, viz. in 1754. This series ("Essays, Physical and Literary") only reached three volumes, and in these the medical papers bear a very small proportion indeed to the others, which have little or no relation to medicine or surgery.

There is a paper in the first volume, however, which is curious, as bearing on a great physiological question agitated of late years, especially by Le Gallois and Dr. Philip. It contains a long series of experiments by the celebrated Dr. Whytt, on the effects of opium on the animal system. It is well known that Le Gallois came to the conclusion that the action of the heart depended on the spinal marrow—while our countryman, Dr. Philip, maintained that it was independent both of the brain and spinal marrow. We shall give one extract from Dr. Philip's experiments, as introductory to a citation from Dr. Whytt.

"The brain of a frog and the spinal marrow as low as the dorsal vertebræ were laid bare. The thorax was then opened, and the heart found acting vigorously; and, from the transparency of its sides, the passage of the blood through it distinctly seen. The part of the spinal marrow which had been laid bare was then removed, but without at all affecting either the motion of the heart, or the passage of the blood through it. The brain was then removed, with the same result.

"*Exp.* 11. The brain and spinal marrow of a frog were removed at the same time. On opening the thorax, the heart was found performing the circulation freely.

"It appears from these experiments that the action of the heart is as independent of every part of the spinal marrow as of the brain; and, consequently, that the opinion of M. le Gallois that it derives its power from that organ, and particularly from the cervical part of it, must be regarded as erroneous." 76.

Let us now hear what Dr. Whytt says.

"Immediately after decollating a frog, I destroyed its spinal marrow, by pushing a small probe down through its spine, which occasioned strong convulsions of all the muscles, especially those of the inferior extremities. Ten minutes after this I opened the thorax, and found the heart beating at the rate of 45 times in a minute. Sixteen minutes after decollation it moved 40 times in a minute. After half an hour it made 36, and, after 50 minutes, only 30 pulsations in the minute, which were now also become very small and feeble." "N.B. When I opened the thorax of another frog, immediately after decollation and destroying its spinal marrow, I observed its heart beating at the rate of 60 in a minute, which is only four or five less than I have generally seen the hearts of frogs make in that time, when their thorax was opened without decollation."* Several other experiments of a similar kind are detailed by Dr. Whytt, in which will be seen an equally curious anticipation and confirmation of some of Dr. Philip's doctrines.

These three volumes, as we before observed, contain a very small proportion of medical papers, and few or none of these of any interest at the present day. We may, however, just glance at one or two facts, before we dismiss the Physical and Literary Essays.

*Remedy for obstructed Menses.* In the year 1755, Dr. A. Hamilton, of Edinburgh had a conversation with his friend, Dr. Hunter, in which the latter proposed compression of the external iliac arteries for the removal of

* Vol. i. p. 282-3.

obstructed menstruation. Dr. Munro was struck with the reasonableness of the proposal, and soon took an opportunity of putting it into execution.

A young girl of 19 years had been obstructed for nearly seven months, in consequence of exposure to cold while menstruating. Her complexion was rather pale and wan—appetite and digestion bad—sickness at stomach—pulse slow and languid. She had no other complaint. Warm semicupia and aloetic medicines were prescribed, and, on the succeeding evening, Dr. H. applied a compress and bandage to the crural arteries, not so tight as to cause complete strangulation of the limbs. The patient was also desired to sit over the steam of warm water. That night she menstruated freely, and continued so to do afterwards.

Considering that other means were used than the mere direction of a greater current of blood than usual to the uterine vessels, we cannot be certain how far this last measure contributed to the object attained. The circumstance, however, is rather curious.

*Tetanus*. Dr. Donald Munro, then physician to St. George's Hospital, communicated a paper on the use of mercury in tetanus, in which he gives the practice of a gentleman in Jamaica, who, in the latter years of his life, saved twelve cases in succession, by placing his patients in a very warm room, and then rubbing in large quantities of mercurial ointment over the limbs and body till ptyalism was raised. This, with large doses of opium, was the only means he used, and by which he was so successful.

Dr. A. Monro, of Edinburgh, after reading this account, tried the same plan in a case of traumatic tetanus, and was also successful.

We must now dismiss the PHYSICAL AND LITERARY ESSAYS, which bring us down to the year 1771 or 2—there being only 8 volumes published in the space of 40 years! What a contrast with the fecundity of the medical periodical press of the present day! As we descend nearer to our own times we shall find a gradual increase of energy and improvement apparent—but more of this in our next number.

### IMPEDIMENTS OF SPEECH.

Among the numerous proposals which have been offered for the removal of Stammering, we have not heard of any so speedy aud effectual as the plan pursued by Dr. Hart, of this metropolis.

The Editor of this Journal was sceptical as to the powers of art, in remedying this imperfection of speech, till one of his own sons, who had laboured under a distressing impediment of this kind for more than ten years, was speedily cured by Dr. Hart, who conducted the process in Dr. Johnson's presence. The rules to be observed are simple and few in number. They are consequently easily learnt and permanently retained in the memory, by which means, the individual is always prepared to check the stammering, when agitation of mind or other circumstance tends to throw him off his guard. Dr. Johnson's son continues free from the impediment, and he has witnessed some other cures equally complete.

*21st December,* 1826.

